# Spiritual Dimension in Neurological and Neurodegenerative Diseases: A Systematic Mapping Review

**DOI:** 10.1007/s10943-022-01683-6

**Published:** 2022-10-15

**Authors:** Rocío de Diego-Cordero, Irene Martos-Lorite, Juan Vega-Escaño

**Affiliations:** 1 Research Group PAIDI-CTS 969 Innovation in HealthCare and Social Determinants of Health, Department of Nursing, Faculty of Nursing, Physiotherapy and Podiatry, University of Seville, Seville, Spain; 2 Department of Nursing, Faculty of Nursing, Physiotherapy and Podiatry, University of Seville, Seville, Spain; 3 Spanish Red Cross Nursing School, University of Seville, Seville, Spain

**Keywords:** Coping, Faith, Quality of life, Neurological/neurodegenerative diseases, Religiosity, Spirituality, Spiritual practices, Well-being

## Abstract

Previous studies have shown the benefits of spirituality/religiosity with regard to health and quality of life for people. The high prevalence of neurological disorders, which are the main diseases that cause disability and dependency around the world, makes neurological disorders especially relevant. This systematic mapping review aimed to map the knowledge of spirituality experienced by people with neurological/neurodegenerative disorders and its influence on outcomes and the ability to cope with the disease. Following specified methodological criteria, a total of 13 articles were selected. The spiritual dimension should be considered a fundamental component of the quality of life and well-being of neurological patients as it can significantly influence their ability to cope with their disease.

## Introduction

Some one billion people worldwide suffer from neurological disorders and more than 6 million people die each year from strokes. It is currently estimated that around 50 million people worldwide suffer from dementia, with nearly 8 million new cases diagnosed each year. (Alzheimer’s is the leading cause of dementia and can contribute to 60–70% of cases.) Similarly, more than 50 million people have epilepsy, and it is estimated that more than 10% of the world population suffers from migraine episodes at some time (World Health Organization, 2006).

The care received by patients with neurological diseases is diverse, there are many neurological treatments, depending on the pathology suffered by the patient. However, the purpose of therapy is comprehensive neurological care that combines the pharmacological treatment of the disease with neurorehabilitation. Neurological rehabilitation is a complex care procedure that therefore requires the existence of a highly specialized and properly trained multidisciplinary team (Vidal-Samsó, 2020).

Spiritual health is fundamentally characterized by an adequate lifestyle, connection with others, asking about the meaning and purpose of life, and transcendence. Furthermore, it directly affects physical, mental, and social health; it can be perceived in the behavior of people, and it can be promoted and improved (Ghaderi et al., 2018).

The attention to spiritual health has gained important recognition in recent times and is considered by many researchers as one of the fundamental aspects of people’s health. Spiritual health has been found to lead to better mental health and positively influence physical health (Ghaderi et al., 2018). Similarly, spirituality is associated with better health, greater well-being, and quality of life, even promoting adaptation to illness and accelerating recovery (Guirao Goris, 2013).

Although it is necessary to address it at any time in life, people’s spiritual needs tend to appear more frequently and even intensify in crisis situations, such as those experienced by a terminal patient, near death, or a critical patient and their families. In this way, spiritual care becomes a fundamental tool to provide comprehensive and humanized care to the patient (de Brito et al., 2013; Ho et al., 2018).

In recent decades, the relationship between spirituality and human health has become an important object of study, reflecting the growing number of published scientific studies that investigate this relationship. In most studies, a positive correlation is shown between the adoption of religious beliefs or spirituality and improved health (Sarrazin Martínez, 2021).

**Fig. 1:**
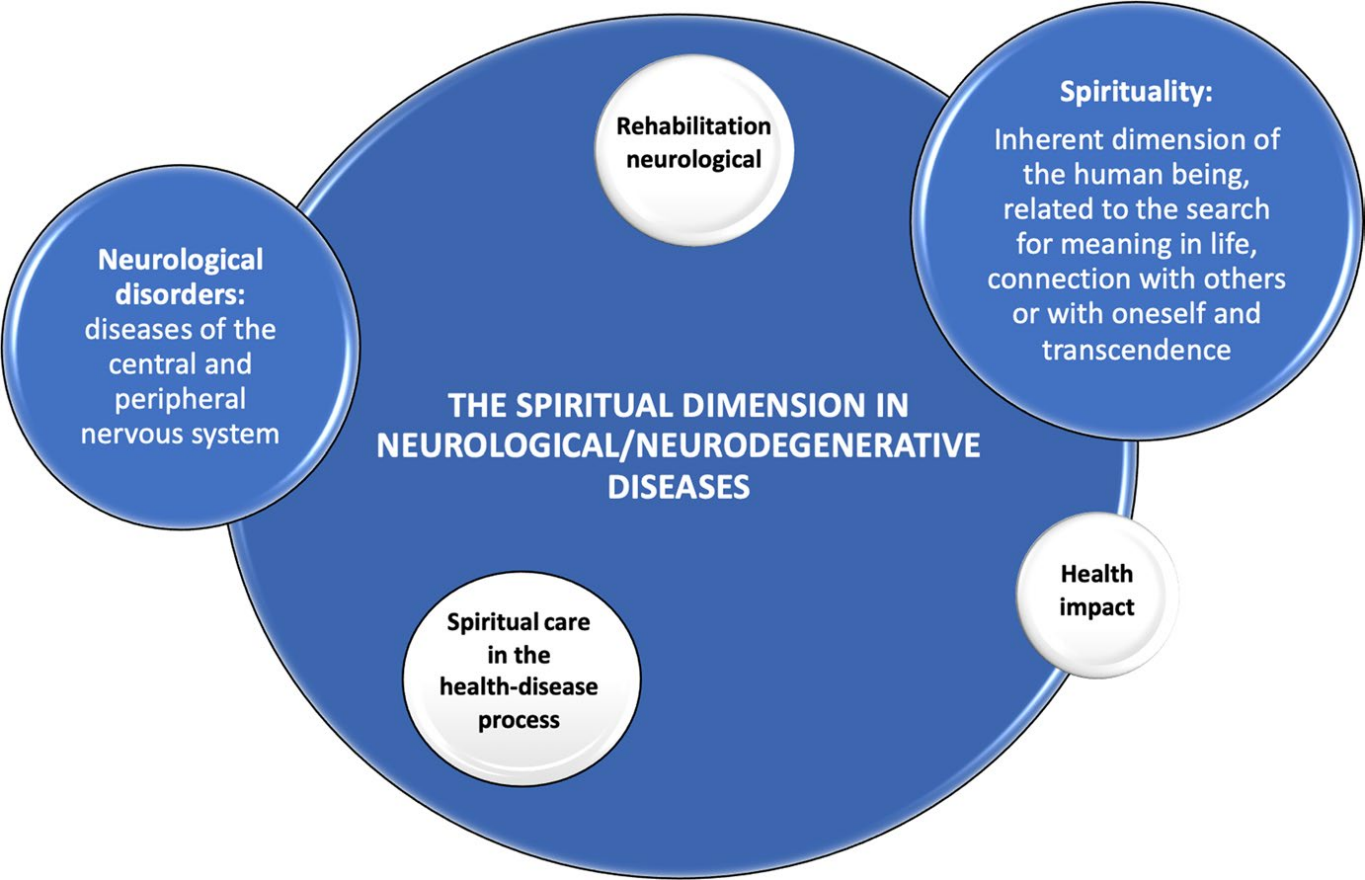
Conceptual framework

Spiritual involvement in coping with various manifestations of chronic disease has been shown to have many benefits. Evidence suggests that spiritual practices help decrease the production of hormones that reduce the number of immune cells and are associated with stress. On the other hand, in addition to promoting a healthier lifestyle, religion helps relieve pain because it increases the number of neurotransmitters involved in this control (Gomes et al., 2019). Furthermore, previous studies have suggested that attending church one or more times a week was associated with lower scores on pain measures (Harrison et al., 2005). Figure 1 summarizes this content and represents the topic of the article in an attention-grabbing way.

However, a terminological confusion about terms “spirituality” and “religiosity” continues. Previous studies have indicated that spirituality and religiosity are related and overlapping, varying according to the cultural context and the dynamic quality of the spirituality itself (de Brito Sena et al., 2021). In addition, this overlap supports the belief that religious traditions should be understood by health professionals, particularly in clinical practice and in the training of health professionals (Puchalski & Larson, 1998).

Previous studies have shown the benefits of spirituality/religiosity in the health and quality of life of people, which, together with the high prevalence of neurological disorders and being the main diseases that cause disability and dependency around the world, make it especially approach relevance.

Makros and McCabe (2003) published an article about research consisting of two studies that aimed to explore the role of religious and spiritual variables in the psychological adjustment and quality of life of people with Multiple Sclerosis (MS). They pointed out that the strongest and most consistent finding in these studies was that the meaning derived from personal belief systems was a strong predictor of psychological adjustment and quality of life. However, until now, few have delved into spiritual care in these situations of illness.

Therefore, this systematic mapping review aimed to map the knowledge of spirituality experienced by people with neurological/neurodegenerative disorders and its influence on results and the ability to cope with the disease.

## Methods

### Search Strategy

A systematic mapping review of the literature was performed between February and April 2022, following the Preferred Reporting Items for Systematic Reviews and Meta-analysis (PRISMA) guidelines for systematic reviews (Page et al., 2021). Due to the fact that registration is not mandatory, the protocol review and review were not registered.

The databases used for this bibliographic review were PubMed, Scopus, and Web of Science. The interface through which the databases were searched was Ovid and the dates of coverage (where this information is provided) were from April 1 to 21, 2022.

The search strategy was designed based on the DeCS/MeSH descriptors, finally using the following descriptors combined with the Boolean operators "AND" and "OR" for its elaboration: *("neurological disorders" or "neurological disease") AND (spirituality or religion or faith or belief system) AND (religion or religious or religiousness or religions or religiosity or spiritual or spirituality or faith)*.

The full line-by-line search strategy run in each database can be seen in Table 1.

**Table 1:** Search strategy

Databases	Search strategy	Query	Selected studies
PubMed	*("Neurological disorders" or "neurological disease") AND (spirituality or religion or faith or belief system) AND (religion or religious or religiousness or religions or religiosity or spiritual or spirituality or faith)*	("Neurological disorders"[All Fields] OR "neurological disease"[All Fields]) AND ("spiritual"[All Fields] OR "spiritualism"[MeSH Terms] OR "spiritualism"[All Fields] OR "spirituality"[MeSH Terms] OR "spirituality"[All Fields] OR "spiritualities"[All Fields] OR "spirituality s"[All Fields] OR "spiritually"[All Fields] OR "spirituals"[All Fields] OR ("religion"[MeSH Terms] OR "religion"[All Fields] OR "religions"[All Fields] OR "religion s"[All Fields]) OR ("faith"[All Fields] OR "faithful"[All Fields] OR "faithfulness"[All Fields] OR "faiths"[All Fields]) OR (("belief s"[All Fields] OR "culture"[MeSH Terms] OR "culture"[All Fields] OR "belief"[All Fields] OR "beliefs"[All Fields]) AND ("drug delivery systems"[MeSH Terms] OR ("drug"[All Fields] AND "delivery"[All Fields] AND "systems"[All Fields]) OR "drug delivery systems"[All Fields] OR "system"[All Fields] OR "system s"[All Fields] OR "systems"[All Fields]))) AND ("religion"[MeSH Terms] OR "religion"[All Fields] OR "religions"[All Fields] OR "religion s"[All Fields] OR ("religious"[All Fields] OR "religiously"[All Fields] OR "religiousness"[All Fields]) OR ("religious"[All Fields] OR "religiously"[All Fields] OR "religiousness"[All Fields]) OR ("religion"[MeSH Terms] OR "religion"[All Fields] OR "religions"[All Fields] OR "religion s"[All Fields]) OR "religiosity"[All Fields] OR ("spiritual"[All Fields] OR "spiritualism"[MeSH Terms] OR "spiritualism"[All Fields] OR "spirituality"[MeSH Terms] OR "spirituality"[All Fields] OR "spiritualities"[All Fields] OR "spirituality s"[All Fields] OR "spiritually"[All Fields] OR "spirituals"[All Fields]) OR ("spiritual"[All Fields] OR "spiritualism"[MeSH Terms] OR "spiritualism"[All Fields] OR "spirituality"[MeSH Terms] OR "spirituality"[All Fields] OR "spiritualities"[All Fields] OR "spirituality s"[All Fields] OR "spiritually"[All Fields] OR "spirituals"[All Fields]) OR ("faith"[All Fields] OR "faithful"[All Fields] OR "faithfulness"[All Fields] OR "faiths"[All Fields]))	22
SCOPUS		ALL ("Cognitive architectures") AND AUTHOR-NAME (smith) TITLE-ABS-KEY (*somatic complaint wom?n) AND PUBYEAR AFT 1993 SRCTITLE (*field ornith*) AND VOLUME (75) AND ISSUE (1) AND PAGES (53–66)	26
WOS		("Neurological disorders" or "neurological disease") AND (spirituality or religion or faith or belief system) AND (religion or religious or religiousness or religions or religiosity or spiritual or spirituality or faith) (All Fields)	48

Two authors (Author 2 and Author 3) independently replicated the search strategy in these three electronic databases and another author (Author 2) reviewed the reference lists of the selected articles, and Author 3 made a review of the gray literature in the Information System on Gray Literature in Europe (OpenGrey). Mendeley software (version 1.19.4) was used for the organization of references in this review.

### Inclusion and Exclusion Criteria for Selected Articles

The eligibility criteria were based on the PICOTS question: P (participant: people with neurological/neurodegenerative disorders), I (intervention: spiritual/religious interventions), C (comparison: between spiritual interventions and waiting list or non-spiritual interventions), O (outcome: the results and coping with the disease), T (time: any follow-up for the intervention), and S (study design: reviews, randomized or non-randomized clinical trials, and literature reviews).

### Study Selection and Data Extraction

After the search, all references were imported via the Mendeley software version 1.19.8. Then, two authors (Author 2 and author 3) independently selected the studies that met the inclusion and exclusion criteria. Initially duplicate records were eliminated, and then, titles and abstracts were reviewed. The discrepancies were resolved by Author 1.

An analysis and synthesis table has also been prepared with the essential information from each included result (Table 2).

**Table 2:** Results

Author Year/Country	Design and sample	Aims	Main results
Giovagnoli et al., 2019/Italy	Quasi-experimental *n* = 199 neurological patients *n* = 66 healthy subjects (control group)	To evaluate spirituality in patients with chronic brain pathologies, with the aim of clarifying its specificity and impact on quality of life	A significantly lower level of spirituality and quality of life was observed in sick patients compared to healthy subjects
Giovagnoli et al., 2009/Italy	Quasi-experimental *n* = 72 neurological patients *n* = 45 healthy subjects (control group)	To explore the role of spirituality in determining quality of life in chronic neurological disorders	The findings support that personal dimensions, such as spirituality, are fundamental components of subjective well-being in chronic neurological patients and that they significantly influence quality of life, even to a greater extent than other variables related to health and disease
McNulty et al., 2004/USA	Cross-sectional descriptive observational *n* = 50 multiple sclerosis patients	To examine the role of spiritual well-being as a mediator and moderator between perceived uncertainty and psychosocial adaptation in multiple sclerosis (MS)	Higher perceived uncertainty and lower spiritual well-being were associated with a worse adaptation to multiple sclerosis, even after demographic variables were considered. Therefore, it is concluded that spiritual well-being positively influences adaptation to multiple sclerosis and also acts to reduce the impact of uncertainty on adaptation
Meyer et al., 2012/USA	Literature review	To assess the efficacy of yoga and its therapeutic benefits in major neurological and psychiatric conditions	Studies show that yoga can be a beneficial complementary treatment for various neurological and psychiatric disorders
Mooventhan & Nivethitha, 2017/India	Literature review	To review the effect of yoga on various neurological disorders	Yoga can be considered an effective aid for patients with neurological diseases such as stroke, Parkinson’s disease, multiple sclerosis, epilepsy, Alzheimer’s disease, dementia, headache, myelopathy, neuropathy
Pretorius & Joubert, 2014/South Africa	Qualitative study *n* = 10 multiple sclerosis patients	To explore the personal experiences of people with multiple sclerosis (MS) in the South African context	Religion turned out to be for the participants one of the main sources of help to cope with multiple sclerosis; along with others such as social support, mobility aids, and having knowledge about the disease
Redfern & Coles, 2015/UK	Literature review	To describe the effects of Parkinson’s Disease (PD) on religious faith and spirituality	An apparent decline in religious and spiritual practices was found in PD as a result of dopaminergic degeneration. On the contrary, the possible positive benefits of disorders such as PD on spiritual development are shown and, vice versa, the possible help in coping with an illness that religious faith can offer
Rogers & MacDonald, 2015/USA	Literature review	To learn about the benefits and potential role of yoga as an alternative treatment for symptom control in people with multiple sclerosis (MS)	The findings support yoga as a safe and effective means of managing MS symptoms
Tedrus & Pereira, 2020/Brazil	Quasi-experimental *n* = 169 adults with epilepsy *n* = 55 healthy subjects (control group)	To analyze the relationship of intrinsic religiosity (IR) with clinical variables of epilepsy, the occurrence of depressive symptoms, and the quality of life of adults with epilepsy	The IR was higher in people with epilepsy compared to the control group. The absence of depressive symptoms was associated with a higher IR. Higher IR was also associated with left hemisphere epileptiform activity and temporal lobe epilepsy
Thygesen et al., 2017/Denmark	Cohort study *n* = 476 patients with chronic neurological disorders	To analyze differences in the incidence of different chronic neurological diseases in a cohort of Danish Adventists and Baptists compared to the general Danish population	The standardized incidence rate (SIR) of dementia or Alzheimer’s disease decreased significantly for members of both religious communities characterized by lifestyle recommendations. However, the SIR of Parkinson’s disease and epilepsy was not significantly different compared to the general population
Vancini et al., 2016/Brazil	Literature review	To know the impact of faith, spirituality, and religiosity as complementary treatments for neurological disorders, especially epilepsy	Spiritual and religious practice can be an active coping and therapeutic strategy to support traditional therapies for people with epilepsy and other neurological disorders
Wade et al., 2018/USA	Cross-sectional descriptive observational study *n* = 354 patients with neurological disorders	To examine the associations between religiosity, spirituality, and happiness in adults living with neurological disorders	Secular or existential spiritual beliefs (SWBS ES) are strongly associated with both the Pemberton Happiness Remembered Index (PHI R) and the Pemberton Experienced Happiness Index (PHI E)
Yamout et al., 2013/Lebanon	Prospective observational study *n* = 201 multiple sclerosis patients	To assess the role of different demographic, clinical, physical, social, economic, and psychological parameters in the perception of patients with multiple sclerosis about their quality of life	Religiosity was identified as one of the main predictors of general quality of life. Other determining factors were depression, social support, level of education, and place of residence, as well as employment and level of fatigue

According to the methods described by Grant and Booth (2009) risk of bias assessment was not conducted for the mapping review.

## Results

A flowchart was done following the PRISMA Declaration (Page et al., 2021). First, 159 articles were retrieved after applying the database search strategy. No results were extracted from the gray literature search.

**Fig. 2:**
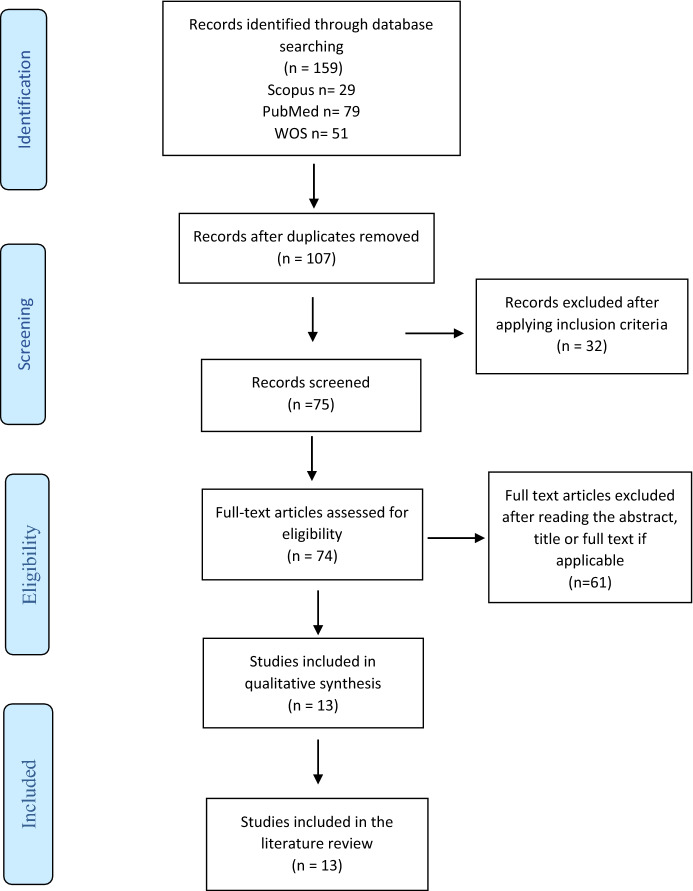
Flowchart

After removing duplicate records and reviewing the titles and abstracts of 107 records, a total of 74 studies were selected for full text reading (Fig. 2).

### Quality and Characteristics Assessment of the Included Studies

A total of 13 articles have been included in the review, which are 3 descriptive observational studies (2 cross-sectional and 1 prospective), 1 cohort study, 3 quasi-experimental studies, 5 literature reviews, and 1 qualitative study.

In addition, 30.8% of the selected results were published in the last 5 years. The English language was predominant, used in all of these. The countries where the most studies were carried out were the USA (30.8%), followed by Italy (15.4%) and Brazil (15.4%).

With regard to the sample size of the included studies, there is a great variety. It found studies from *n* = 10, such as the one by Pretorius and Joubert (2014), to others such as the one by Thygesen et al., (2017) with *n* = 476.

The instruments used in studies were different: the Quality of Life of the World Health Organization (WHOQOL 100); Spirituality, Religiousness, and Personal Beliefs (SRPB); Beck Depression Inventory (BDI); State-Trait Anxiety Inventory (STAI); Multi-Agility Self-Report Questionnaire (MASQ) (Giovagnoli et al., 2009, 2019); Mishel Uncertainty in Illness Scale (MUIS) (McNulty et al., 2004); Neurological Disorders, Depression Inventory for Epilepsy (NDDI-E, Intrinsic Religiosity Inventory (IRI), Brief Cognitive Battery-Edu (BCB-Edu), Inventory of quality of life in epilepsy (QOLIE-31) (Tedrus & Pereira, 2020); the Pemberton Happiness Index (PHI), the Short Questionnaire to Assess Health-Enhanced Physical Activity (SQUASH), the Wechsler Test of Adult Reading (WTAR), the Wechsler Abbreviated Scale of Intelligence—Second Edition (WASI-II), Depression Visual Analog Scale (Depression VAS) (Wade et al., 2018); and Multiple Sclerosis Quality of Life-54 (MSQOL-54), Hamilton Depression Rating Scale (HDRS), Fatigue Severity Scale (FSS), Brief Pain Inventory Average Pain Score (BPI) (Yamout et al., 2013). Only the Spiritual Well-Being Scale (SWB) was used in two studies (McNulty et al., 2004; Wade et al., 2018).

Almost half of the articles selected for this review investigated the impact of spirituality on the quality of life and well-being of patients with neurological diseases (Giovagnoli et al., 2009, 2019; Tedrus & Pereira, 2020; Wade et al., 2018; Yamout et al., 2013). Similarly, there were articles that focused on investigating the role and effects of performing spiritual practices such as yoga in patients with a neurological disorder (Meyer et al., 2012; Mooventhan & Nivethitha, 2017; Rogers & MacDonald, 2015), while others also explored spirituality/relativity as a possible aid in adaptation and coping with neurological disease (McNulty et al., 2004; Pretorius & Joubert, 2014; Redfern & Coles, 2015; Vancini et al., 2016). Finally, a study established the objective of comparing the incidence of some neurological diseases in people with religious beliefs compared to the general population (Thygesen et al., 2017).

### Impact of Spirituality on the Quality of Life and Well-Being of Neurological Patients

The included studies have related religiosity/spirituality to quality of life and well-being, dealing with aspects such as happiness and intrinsic religiosity. In this regard, Giovagnoli et al., (2009) and Giovagnoli et al., (2019) explored the role of spirituality in determining the quality of life of patients with chronic neurological disorders. They observed that spirituality, together with other personal dimensions, is a fundamental component of subjective well-being in chronic neurological patients and that it significantly influences their quality of life.

However, Wade et al., (2018) investigated the association between religiosity, spirituality, and happiness in 354 outpatients with neurological disorders in the USA. They determined that spiritual beliefs, specifically secular or existential, are strongly associated with higher levels of happiness in these patients.

Tedrus and Pereira (2020) investigated the relationship between intrinsic religiosity and the appearance of depressive symptoms and the quality of life of people with epilepsy. The absence of depressive symptoms was associated with greater religiosity. Furthermore, religiosity was found to be higher in people with epilepsy compared to the control group.

Finally, Yamout et al., (2013) conducted a prospective observational study that included 201 patients with multiple sclerosis in Lebanon. These authors identified religiosity as one of the main factors influencing the quality of life of these patients. Other determining factors they observed were depression, social support, level of education, and place of residence, as well as employment and level of fatigue.

### The Role of Spirituality in Adaptation, Coping and Incidence of Neurological Disease

Regarding the role that spirituality plays in the adaptation of included studies, included studies address aspects such as spiritual well-being, uncertainty, and religious faith as coping mechanisms.

In this sense, spiritual well-being positively influences adaptation to illness and also works by reducing the impact of uncertainty on adaptation (McNulty et al., 2004). Likewise, a qualitative study exploring the personal experiences of people with MS in South Africa found that religion was for participants one of the main resources that helped them cope with the disease, because according to these patients, it helped them with acceptance, gave them purpose in life, and even hope for healing (Pretorius & Joubert, 2014).

Redfern and Coles (2015) reviewed research studying the effects of Parkinson’s disease (PD) on religious faith and spirituality. Possible benefits of disorders such as PD on spiritual development and, vice versa, possible help in coping with the illness that religious faith can offer were identified. Similarly, Vancini et al., (2016) highlighted the impact of faith, spirituality, and religiosity as complementary treatments for neurological disorders, especially epilepsy. They found that spiritual and religious practice can be an active coping and therapeutic strategy to support traditional therapies for people with epilepsy and other neurological disorders.

This impact has also been studied in the opposite direction, focusing on the incidence of neurological disorders in patients with religious/spiritual beliefs compared to those who do not. In this regard, Thygesen et al., (2017), in their study that included 476 patients with chronic neurological disorders (303 Seventh-day Adventists and 173 Baptists) in Denmark, showed that the standardized incidence rate (SIR) of dementia or Alzheimer’s disease decreased significantly for members of both religious communities characterized by lifestyle recommendations. However, the SIR of Parkinson’s disease and epilepsy were not significantly different compared to the general population.

### Efficacy and Effects of Performing Spiritual Practices in Patients with Neurological Disorders

In relation to the clinical application of spiritual practices in patients with neurological disorders, the included studies focus on the practice of yoga as a spiritual technique. Meyer et al., (2012) found that of seven randomized controlled trials of yoga in patients with neurological disorders, six found significant positive effects and of thirteen randomized controlled trials of yoga in patients with psychiatric disorders, ten found significant positive effects.

In this regard, other reviews on the effect of yoga on various neurological disorders agree that yoga can be considered an effective help for these patients, since positive effects of its practice have been identified as part of the treatment. Therefore, the findings show that yoga can be a safe and effective means of managing the symptoms of multiple sclerosis (Mooventhan & Nivethitha, 2017; Rogers & MacDonald, 2015).

## Discussion

This systematic mapping review mapped the knowledge of the spirituality experienced by people with neurological/neurodegenerative disorders and its influence on outcomes and the ability to cope with the disease.

The studies included in this review have indicated that spirituality/religiosity is closely related to better quality of life and emotional well-being in patients suffering from some type of neurological disorder. In turn, other research shows the importance of the spiritual dimension in the adaptation and coping with the disease, in this case neurological; consider the religious faith as a resource that can help these patients accept and cope with this condition. One of the studies also reflected a lower incidence of some neurological disorders in patients with strong religious beliefs and practices compared to the general population; however, regarding other diseases of this type, there were no significant differences in the incidence of one group compared to the other. On the other hand, some spiritual practices, such as yoga, have been shown to have positive effects and to be an effective aid in the treatment of some neurological disorders.

Regarding the impact of spirituality/religiosity on the quality of life and well-being of neurological patients, Wade et al., (2018) noted a unique association between spiritual beliefs, specifically secular or existential, with higher levels of happiness in neurologically ill patients. Similarly, the study by Tedrus and Pereira (2020) carried out in Brazil, in which 169 people with epilepsy and 55 healthy people who made up the control group participated, identified a greater intrinsic religiosity in patients with epilepsy compared to the control group. Furthermore, in the first group, the absence of depressive symptoms was associated with greater religiosity.

Regarding this issue, previous studies have shown various benefits of spirituality/religiosity on mental health (Sarrazin Martínez, 2021). Smith (2003) analysis pointed out that religiosity can reduce the individual’s vulnerability to symptoms of depression and anxiety, while the results of the study by Cano García and Quintero Nuñez (2020), carried out on a sample of 306 participants belonging to three religious groups, determined a correlation between spirituality and/or religiosity with mental health and psychological well-being.

In Lebanon, Yamout et al., (2013) conducted a study with a sample of 201 patients with multiple sclerosis, identifying religiosity as one of the determining factors in the perception these patients had of their quality of life. In this sense, the findings of Giovagnoli et al., (2009) also agree, since they maintain that personal dimensions, such as spirituality, are fundamental components of subjective well-being in chronic neurological patients and that they significantly influence their quality of life, even to a greater extent than other variables related to health and safety disease. In turn, the study by Giovagnoli et al., (2019), who evaluated spirituality in patients with chronic brain pathologies, with the aim of clarifying its specificity and position in a multidimensional model of quality of life, obtained similar results.

These findings are consistent with other research that emphasizes the role of spirituality in determining the psychological well-being of people with other diseases. For example, Brandão et al., (2021) concluded that there is a positive association between spirituality/religiosity and quality of life in women with breast cancer who receive radiotherapy. Likewise, in another study conducted in outpatients with heart failure, greater spiritual well-being, specifically sense of life/peace, was closely related to fewer depressive symptoms and therefore a better perceived quality of life (Bekelman et al., 2007).

In relation to the role of spirituality in adaptation, coping, and incidence of neurological disease, the study by McNulty et al., (2004) in a sample of 50 patients with multiple sclerosis (SM) showed that spiritual well-being significantly influences adaptation to the disease, and also acts to reduce the impact of uncertainty on this adaptation. Similarly, the findings of another study whose objective was to explore the personal experiences of people with SM in the South African context showed that religion turned out to be for participants one of the main resources that helped them cope with this condition effectively, since it was useful for them with acceptance of their condition, giving them a purpose in life and even hope of healing (Pretorius & Joubert, 2014). Redfern and Coles (2015) and Vancini et al., (2016), in their reviews, also agree on religious faith as a possible help in coping with illness, specifically Parkinson’s disease and epilepsy.

Neuropsychiatric signs and symptoms are among the most common non-motor features of Parkinson’s disease, with anxiety and depression being the most frequent manifestations (Weintraub et al., 2022). This research determines that psychosocial factors can play an important role in the control and prevention of these symptoms. Some of the protective factors are, for example, spiritual well-being, social support, access to multidisciplinary health services, and maintenance of employment, while negative thoughts about the diagnosis, the course of the disease and its social consequences as well as the patient’s inability to face the challenges related to Parkinson’s disease could be a risk factor for its appearance.

On the other hand, a study whose objective was to explore the psychosocial aspects that influence coping with Parkinson’s patients and their family caregivers identified three main aspects in which the study participants agreed: functionality of health care; family environment and acceptance of the disease, highlighting the need for care provided to these patients to have a comprehensive approach, which, in addition to symptom control, addresses the psychosocial aspects that affect coping with the disease (Navarta-Sánchez et al., 2017).

In recent decades, there has been an increase in scientific interest in spirituality/religion as a coping strategy. The search for religious and/or spiritual support is especially necessary in situations of crisis or threat, such as those that a terminal patient, in a critical situation, or with a chronic disease can experience (de Brito Sena et al., 2021). Therefore, studies such as the one by Landa-Ramírez et al., (2017) determined that spiritual and/or religious coping is present in the Latino population with breast cancer, especially in elderly women, providing them with emotional and social support during their illness. Similarly, the results of another study focused on chronic disease in the elderly showed that religiosity/spirituality/faith positively interferes in addressing life’s obstacles and difficulties, strengthening the patient’s resilience, thus improving their quality of life (Rocha & Ciosak, 2014).

Lucchetti et al., (2021), in their research, also highlight the role of spirituality in coping with adverse situations. The study in question was conducted in Brazil with a sample of 485 participants, with the aim of investigating the association between spirituality/relativity and the mental health consequences of social isolation during the COVID-19 pandemic. The findings indicated that there was a high use of religious and spiritual beliefs during the pandemic, which was associated with better health outcomes, as higher levels of hope, as well as lower levels of fear, worry, and sadness, were identified in the most religious participants and with greater spirituality.

Specifically referring to the incidence of neurological disorders in patients with religious/spiritual beliefs, the Danish study by Thygesen et al., (2017) stands out, pointing out that the incidence of dementia or Alzheimer’s disease decreased significantly for members of religious communities characterized by lifestyle recommendations. Therefore, various scientific investigations agree that the keys to preventing many chronic neurodegenerative diseases are found in lifestyle factors. For example, lifestyle, especially eating habits and physical activity, has been observed to play an important role in preventing the onset of Alzheimer’s disease and other neurodegenerative diseases (Flicker, 2010; López Yes et al., 2021; Yang et al., 2021). Similarly, in the case of cerebrovascular disease, Ruiz-Sandoval et al., (2010) determined that healthy lifestyles are essential in the primary and secondary prevention of this disease, in addition to reducing severity and improving prognosis when it occurs. On the contrary, unfavorable lifestyle habits, which are highly prevalent among the population, increase the risk of suffering a stroke.

Regarding the efficacy and effects of the practice of spiritual practices in patients with neurological disorders, it should be noted that the articles obtained that address this issue focus on the practice of yoga. In general, the data provided in these reviews agree that yoga can be considered an effective aid for patients with some neurological diseases, since the positive effects of its practice have been evidenced as part of treatment (Meyer et al., 2012; Mooventhan & Nivethitha, 2017; Rogers & MacDonald, 2015).

Regarding other spiritual mind–body therapies, Bajaj et al., (2017) evaluated the efficacy of meditation in patients with end-stage liver disease and their caregivers. After 4 meditation sessions, the study participants experienced less depression, less burden, and better sleep hygiene for their caregivers. Similarly, Badenes and Ausín (2021) show that interventions aimed at older people with chronic disease, including mindfulness components, achieve improvements in variables such as depression, anxiety, acceptance, and intensity of pain, subjective decrease in pain, subjective improvement of the ability to maintain attention in the present moment, subjective improvement in sleep, subjective well-being, and quality of life. On the other hand, a yoga mindfulness program guided by self-transcendence theory, in which 138 patients with Parkinson’s disease participated, was found to be as effective as stretching exercises and resistance training in improving motor dysfunction and memory loss mobility, with the added benefits of significant improvement in both anxiety and depression, and increased spiritual well-being and quality of life related to health (Kwok et al., 2019).

### Strengths and Limitations

This study has important strengths. This mapping systematic review has allowed knowledge to be updated, and the identification of gaps in the evidence base, and it has been a tool to identify knowledge gaps and new research questions.

However, this study has several limitations that must also be taken into account. The search has been carried out in only three databases (PubMed, Scopus, and WOS), which has prevented access to all articles that may have been carried out in relation to the research question posed.

Another limitation is related to the measurement of variables such as quality of life, well-being, or coping, because although there are scales and measurement instruments, these, in turn, are determined by different clinical, physical, psychosocial, or demographic variables, so that do not depend solely on the spiritual factor. In this way, although most of the studies that investigated this relationship describe that these variables were considered, in others they were not indicated in the studies.

Finally, no evaluation of the quality of the included review studies has been performed, which limits their value in making recommendations in clinical practice.

## Conclusions

Spirituality in patients with a neurological disorder seems to be closely related to a higher quality of life and emotional well-being.

The spiritual dimension is an important aspect in adaptation and coping with neurological diseases, considering religious faith as a resource that can help these patients accept and cope with their condition.

There is a lower incidence of some neurological disorders in patients with strong religious beliefs and practices compared to the general population, with the lifestyles recommended by certain religions being a protective factor against the development of certain diseases and health problems.

In addition, spiritual practices such as yoga have been shown to have positive effects and to be an effective aid in the treatment of some neurological disorders. However, it is necessary to take the appropriate measures to avoid possible adverse effects when performing advanced yoga practices.

### Implications for Clinical Practice

These findings have important implications for clinical practice. In the present review, spirituality has been identified as an important aspect in the adaptation and coping with neurological diseases. This is particularly important for healthcare management, as they should be aware of the importance of this dimension for professional health. Providing spiritual support could be important strategies to incorporate into healthcare settings.

Spiritual dimension is considered a fundamental component of the quality of life and well-being of neurological patients and significantly influences their ability to cope with the disease.

For this reason, it is necessary that the care provided to these patients has a comprehensive approach that, in addition to the control of symptoms, addresses personal aspects such as spirituality.

Finally, the lack of randomized controlled trials in this review draws attention to the lack of solid evidence that spiritual and religious interventions were capable of modifying the approach of neurological diseases. More studies are definitely needed to incorporate the consolidated observational findings into clinical practice.
